# Establishment of prognostic model for postoperative patients with metaplastic breast cancer: Based on a retrospective large data analysis and Chinese multicenter study

**DOI:** 10.3389/fgene.2022.993116

**Published:** 2022-08-25

**Authors:** Ge Wang, Xiaomin Sun, Xin Ren, Mengmeng Wang, Yongsheng Wang, Shukun Zhang, Jingye Li, Wenping Lu, Baogang Zhang, Pingping Chen, Zhiqiang Shi, Lijuan Liu, Jing Zhuang

**Affiliations:** ^1^ Clinical Medical Colleges, Weifang Medical University, Weifang, China; ^2^ Shandong Cancer Hospital and Institute, Shandong First Medical University and Shandong Academy of Medical Sciences, Jinan, China; ^3^ Department of Pathology, Weihai Municipal Hospital, Cheeloo College of Medicine, Shandong University, Weihai, China; ^4^ Department of Oncology, Linyi Central Hospital, Linyi, China; ^5^ Department of Oncology, Guang’ Anmen Hospital, China Academy of Chinese Medicine Sciences, Beijing, China; ^6^ Department of Pathology, Weifang Medical University, Weifang, China; ^7^ Department of Pathology, The People’s Hospital of Rizhao, Rizhao, China; ^8^ Department of Oncology, Weifang Traditional Chinese Hospital, Weifang, China

**Keywords:** metaplastic breast cancer, multicenter, nomogram, postoperative, overall survival

## Abstract

**Purpose:** Models for predicting postoperative overall survival of patients with metaplastic breast cancer have not yet been discovered. The purpose of this study is to establish a model for predicting postoperative overall survival of metaplastic breast cancer patients.

**Methods:** Patients in the Surveillance, Epidemiology, and End Results database diagnosed with MBC from 2010 to 2015 were selected and randomized into a SEER training cohort and an internal validation cohort. We identified independent prognostic factors after MBC surgery based on multivariate Cox regression analysis to construct nomograms. The discriminative and predictive power of the nomogram was assessed using Harrell’s consistency index (C-index) and calibration plots. The decision curve analysis (DCA) was used to evaluate the clinical usefulness of the model. We verify the performance of the prediction model with a Chinese multi-center data set.

**Results:** Multifactorial analysis showed that age at diagnosis, T stage, N stage, M stage, tumor size, radiotherapy, and chemotherapy were important prognostic factors affecting OS. The C-index of nomogram was higher than the eighth edition of the AJCC TNM grading system in the SEER training set and validation set. The calibration chart showed that the survival rate predicted by the nomogram is close to the actual survival rate. It has also been verified in the SEER internal verification set and the Chinese multi-center data set.

**Conclusion:** The prognostic model can accurately predict the post-surgical OS rate of patients with MBC and can provide a reference for doctors and patients to establish treatment plans.

## Background

Metaplastic breast cancer (MBC) is a rare subtype of breast cancer, with unique histopathological and molecular characteristics (McCart Reed et al., 2019), accounting for less than 1% of all breast malignancies (Haque et al., 2018). While most human cancers have only one histological component in a primary tumor, MBC is a heterogeneous cancer with diverse shapes and two or more different tissue types (Avigdor et al., 2017), including pure epithelial components (e.g., squamous cells), mesenchymal components (e.g., spindle cells, chondroids, osseous and myoid cells), or a mixture of the two (Jung et al., 2010). In 2000, the World Health Organization (WHO) recognized MBC as a unique pathological entity, since then, the incidence of MBC has increased (Pezzi et al., 2007; Drekolias and Mamounas, 2019).

MBC patients are not sensitive to chemotherapy and hormone therapy, and relevant targeted therapy and immunotherapy programs have not yet been developed. Surgery has often been the mainstay of therapy, and the role of multimodal therapy remains an area of active investigation (Ong et al., 2018). MBC is characterized by strong invasiveness, poor prognosis, and is often negative in estrogen receptor (ER), progesterone receptor (PR), and human epidermal growth factor receptor 2 (HER2) tests; its prognosis is worse than that of triple-negative breast cancer, and the survival rate is lower (Bae et al., 2011; El Zein et al., 2017). The mortality rate of breast cancer is gradually increasing, and metaplastic breast cancer also plays a role in it.

At present, there are no standardized guidelines for MBC, and most clinicians still guide the treatment of MBC according to the scheme for invasive ductal cancer (IDC) (Pezzi et al., 2007). However, compared with IDC, MBC has a larger tumor size, lower degree of differentiation, faster growth rate, and less axillary lymph node metastasis (Jung et al., 2010; Nelson et al., 2015; Bian et al., 2016). Since they have different clinicopathological features, it may be unreasonable to predict the pathological course and prognosis of MBC from existing studies on patients with IDC (Zhang et al., 2018; Han et al., 2019). Therefore, MBC requires independent treatment and management strategies and its own customized model for personalized evaluation of postoperative survival of patients.

The TNM staging system proposed by the American Joint Committee on Cancer (AJCC) is a common tool used by oncologists to predict disease progression and design treatment strategies. However, considering that there are many factors affecting cancer progression, it may be unreasonable to predict the prognosis of MBC based on TNM staging alone (Chen et al., 2019). In this respect, the nomogram makes up for its shortcoming (Iasonos et al., 2008). Due to the low incidence of MBC, most of the studies on MBC come from a single medical institution or a limited cohort of case reports (Leyrer et al., 2017; Han et al., 2019). Therefore, based on data from the Surveillance, Epidemiology, and End Results (SEER) large-scale database in the United States, and the clinical and pathological data of patients with MBC from six large hospitals in Shandong Province, China, we explored the indicators that affect the prognosis of patients with MBC and constructed a model to predict survival. The objective is to provide a reference for informing the treatment of patients with MBC.

## Materials and methods

### Patient selection and data processing

Patient data from 2010 to 2015 were screened from the SEER database (1975–2016 varying) using SEER*Stat version 8.3.8 (https://seer.cancer.gov/) and six large hospitals in Shandong, China. The obtained data were divided at random into a training set and an internal verification set in a 7:3 ratio. Based on clinical experience, existing literature, and the availability of information in the SEER data, the following variables were selected and evaluated: age, race, marital status (married: married/unmarried or domestic partner; single: unmarried; others: divorced/separated/widowed), grade, tumor size, laterality, T stage, N stage (negative, positive), M stage, subtype, ER status, PR status, HER2 status, type of surgery (lumpectomy, mastectomy), radiation (no/unknown, yes), and chemotherapy (no/unknown, yes).

The inclusion criteria for data screening were:

a) Women with primary MBC;
b) MBC diagnosis was consistent with the International Classification of Disease for Oncology third edition (coded as 8032/3, 8033/3, 8070/3, 8570/3–8572/3, 8575/3, 8980/3, 8982/3);
c) Primary site: C50.0–C50.6, C50.8, C50.9.

The exclusion criteria were:

a) Incomplete clinical pathological data or unknown records,
b) Histological diagnosis was not positive,
c) Survival time was less than 1 month or unknown,
d) Patients with bilateral MBC
e) The patient did not undergo surgery at the primary tumor site.

For external verification, a Chinese multicenter validation set based on patients diagnosed between January 2010 and December 2020 from six hospitals (Shandong Cancer Hospital, Weihai Municipal Hospital, Rizhao People’s Hospital, Linyi Central Hospital, Weifang Traditional Chinese Medicine Hospital, and the affiliated Hospital of Weifang Medical College) was included in the study. The inclusion/exclusion criteria for the Chinese multicenter data were consistent with that of the SEER dataset. Overall survival (OS) was used as the endpoint of this study and defined as the time from diagnosis to death of any cause or to the last follow-up. The last day of follow-up was 1 December 2020. For the retrospective analysis of the external validation set, we received approval by the ethics committee of the Weifang Traditional Chinese Hospital in Shandong, China. All patient data were used anonymously.

### Prediction model was built based on multivariate analysis

In the training set, a multivariate Cox proportional hazard regression model was used to further analyze the prognostic variables identified from univariate analysis that were significantly related to postoperative OS in patients with MBC. The results are reported using hazard ratio (HR) and 95% confidence interval (CI). Based on the results of multivariate analysis, a nomogram was constructed to predict the probability of OS at 1, 3, and 5 years in patients with MBC.

### Discrimination and calibration of the nomogram

We used the C-index, receiver operating characteristic curve (ROC), and area under the curve (AUC) to evaluate the distinguishing capabilities of the prediction model. A higher C-index value indicates a better discriminative performance by the nomogram. Calibration was evaluated such that how close the calibration curve was to the standard curve indicated the predictive ability of the model. To test the accuracy and reliability of the nomogram, the SEER internal verification set and Chinese multi-center external data set were used. To evaluate the effectiveness of the training and internal validation sets, the nomogram was compared with the eighth edition AJCC staging model.

### Survival risk analysis and clinical usefulness

The patients were divided into high-risk and low-risk groups according to the risk score obtained by the nomogram. The Kaplan-Meier method was used to assess the significance of survival differences between the two risk groups. The difference in survival between a triple negative metaplastic breast cancer (TNMC) group and a non-triple negative metaplastic breast cancer (NTNMC) group was also analyzed. Whether radiotherapy would bring survival benefits to elderly female patients was also studied. The DCA was used to evaluate the clinical usefulness of the nomogram.

### Statistical analysis

All statistical analyses were performed using SPSS 25.0 (SPSS Inc., Chicago, IL, United States) and R software (version 4.0.3; http://www.r-project.org/). X-Tiles (version 3.6.1, Yale University, New Haven, CT, United States) was used to determine the best cut-off value for age and tumor size. Categorical variables were analyzed as frequencies and proportions. Cox proportional risk regression analysis was performed using SPSS. The nomogram, ROC curve and calibration curve were generated using software packages (“foreign,” “survival,” “rms”); The DCA was performed using the R package (“stdca.R”). A *p* value < 0.05 was considered statistically significant.

## Results

### Patient characteristics

**FIGURE 1:**
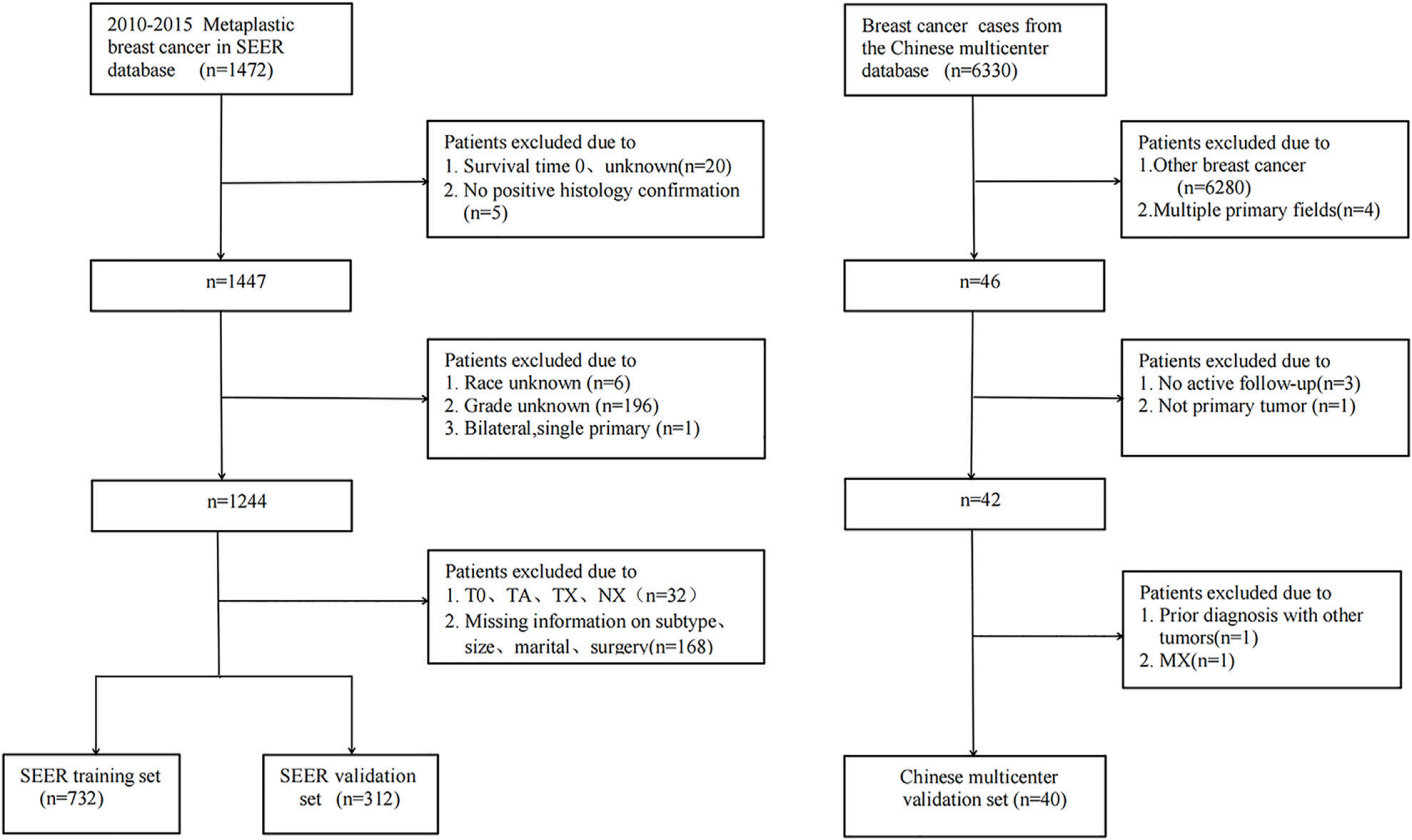
Flow diagram of MBC patient selection for the study.

**FIGURE 2:**
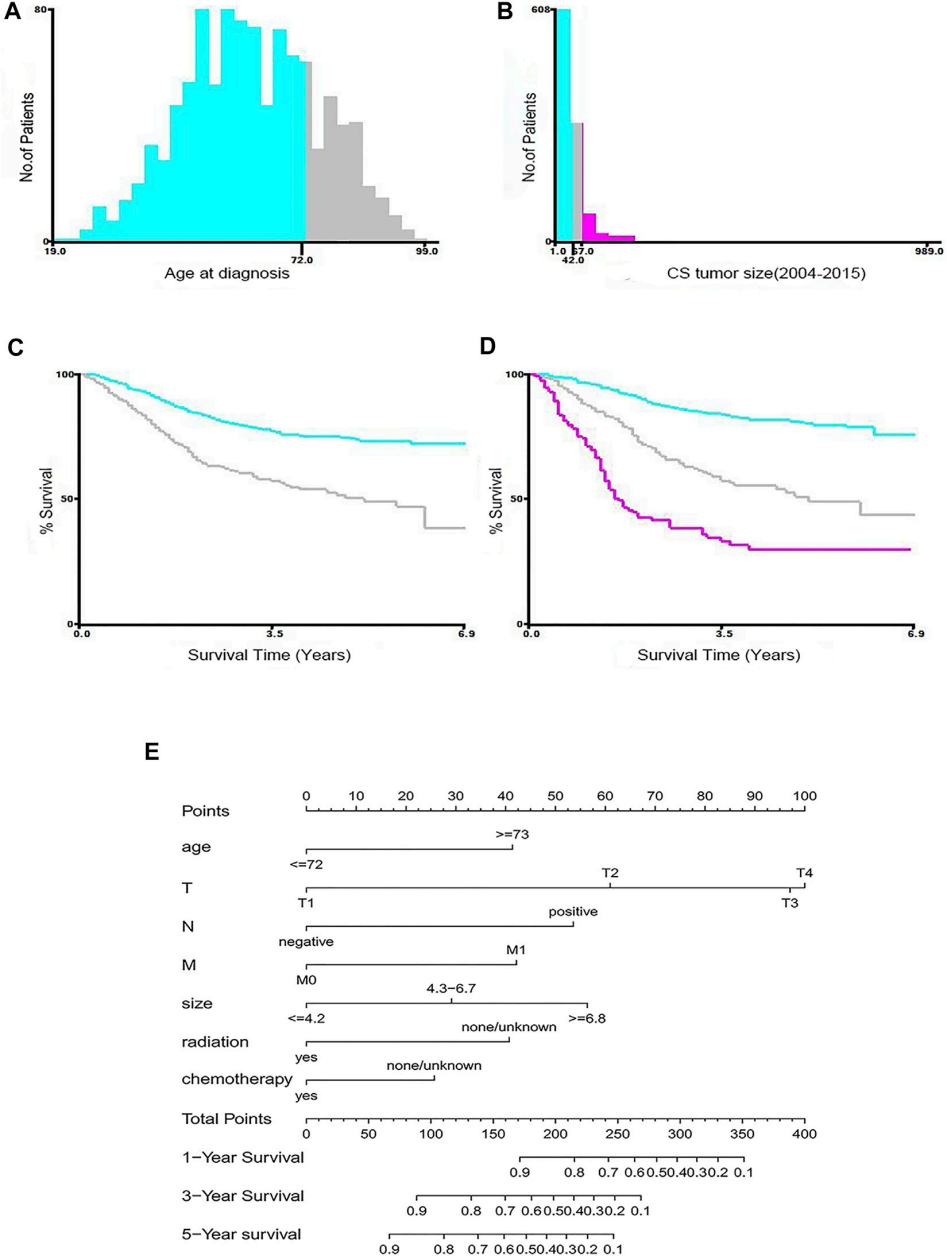
Identification of optimal cut-off values for age **(A,C)** and tumor size **(B,D)** by X-tile software analysis. The optimal cut-off value of age is identified as 72-years, and the cut-off for tumor size is identified as 4.2, 6.7 cm based on entire cohort. Nomograms for predicting the 1-, 3-, and 5-years OS rates of patients with MBC **(E)**.

As shown in Figure 1, 1,044 patients with postoperative MBC were screened and divided into a training set (n = 732) and internal validation set (n = 312). As an external validation dataset, 40 eligible patients from a Chinese multicenter study were included. The best cut-off for age was 72 years, and the tumor size was 4.2, 6.7 cm (Figures 2A–D). Table 1 summarizes the demographic and clinicopathological characteristics of the training, internal validation, and complete cohorts. In the training set, the median survival time was 31 months (interquartile range [IQR]: 18–54 months), and the median patient age was 61 years (IQR: 51–72 years). Table 1 shows the data distribution for the multiple centers in China. Among the patients, the median survival time was 41.5 months (IQR:13–56.75 months), the median age was 52.5 years (IQR: 46–61 years).

**TABLE 1:** Patients’ demographics and clinicopathological characteristics.

Variables	All patients (n = 1,044) N (%)	SEER training set (n = 732) N (%)	SEER validation set (n = 312) N (%)	Chinese multicenter validation set (n = 40) N (%)
Age				
≤72	788 (75.5)	554 (75.7)	234 (75.0)	39 (97.5)
≥73	256 (24.5)	178 (24.3)	78 (25.0)	1 (2.5)
Race				
Black	170 (16.3)	119 (16.3)	51 (16.3)	--
White	796 (76.2)	566 (77.3)	230 (73.7)	--
Other	78 (7.5)	47 (6.4)	31 (9.9)	--
Marital status				
Married	575 (55.1)	409 (55.9)	166 (53.2)	--
Single	169 (16.2)	113 (15.4)	56 (17.9)	--
Other	300 (28.7)	210 (28.7)	90 (28.8)	--
Laterality				
Left	533 (51.1)	378 (51.6)	155 (49.7)	--
Right	511 (48.9)	354 (48.4)	157 (50.3)	--
Grade				
I	24 (2.3)	18 (2.5)	6 (1.9)	--
II	120 (11.5)	79 (10.8)	41 (13.1)	--
III	863 (82.7)	609 (83.2)	254 (81.4)	--
IV	37 (3.5)	26 (3.6)	11 (3.5)	--
8th AJCC T stage				
T1	254 (24.3)	183 (25.0)	71 (22.8)	7 (17.5)
T2	514 (49.2)	347 (47.4)	167 (53.5)	19 (47.5)
T3	187 (17.9)	134 (18.3)	53 (17.0)	11 (27.5)
T4	89 (8.5)	68 (9.3)	21 (6.7)	3 (7.5)
8th AJCC N stage				
negative	800 (76.6)	563 (76.9)	237 (76.0)	34 (85)
positive	244 (23.4)	169 (23.1)	75 (24.0)	6 (15)
8th AJCC M stage				
M0	1,001 (95.9)	700 (95.6)	301 (96.5)	38 (95)
M1	43 (4.1)	32 (4.4)	11 (3.5)	2 (5)
Tumor size				
≤4.2	700 (67.0)	486 (66.4)	214 (68.6)	25 (62.5)
4.3–6.7	215 (20.6)	148 (20.2)	67 (21.5)	4 (10.0)
≥6.8	129 (12.4)	98 (13.4)	31 (9.9)	11 (27.5)
Subtype				
Luminal A	252 (24.1)	175 (23.9)	77 (24.7)	--
Luminal B	24 (2.3)	17 (2.3)	7 (2.2)	--
HER2 enriched	41 (3.9)	33 (4.5)	8 (2.6)	--
Triple negative	727 (69.6)	507 (69.3)	220 (70.5)	--
ER				
Positive	212 (20.3)	144 (19.7)	68 (21.8)	--
Negative	832 (79.7)	588 (80.3)	244 (78.2)	--
PR				
Positive	139 (13.3)	98 (13.4)	41 (13.1)	--
Negative	905 (86.7)	634 (86.6)	271 (86.9)	--
HER2				
Positive	65 (6.2)	50 (6.8)	15 (4.8)	--
Negative	979 (93.8)	682 (93.2)	297 (95.2)	--
Radiation				
No/unknown	540 (51.7)	378 (51.6)	162 (51.9)	30 (75)
Yes	504 (48.3)	354 (48.4)	150 (48.1)	10 (25)
Chemotherapy				
No/unknown	324 (31.0)	231 (31.6)	93 (29.8)	9 (22.5)
Yes	720 (69.0)	501 (68.4)	219 (70.2)	31 (77.5)
Surgery type				
Lumpectomy	423 (40.5)	291 (39.8)	132 (42.3)	8 (20)
Mastectomy	621 (59.5)	441 (60.2)	180 (57.7)	32 (80)

### Prognostic factors associated with OS in patients with metaplastic breast cancer

Table 2 summarizes the results of univariate and multivariate Cox regression analyses in the training cohort. Univariate regression analysis showed that age at diagnosis, tumor size, marital status, grade, T stage, N stage, M stage, radiotherapy, chemotherapy, and type of surgery were significantly correlated with OS; while race, laterality, subtype, ER status, PR status, and HER2 status were not. Multivariate analysis identified that age at diagnosis, T stage, N stage, M stage, radiation, chemotherapy, and tumor size were independent predictors of survival. The drawn survival curve also shows that these variables are independent prognostic factors for MBC (Supplementary Figure S1A–G). Based on the prognostic factors selected from the training cohort, a nomogram was developed to predict the 1-, 3-, and 5-years survival probability of patients with MBC (Figure 2E).

**TABLE 2:** Univariate and multivariate Cox regression analysis based on all variables in the training set.

Variables	Univariate analysis			Multivariate analysis	
	HR (95% CI)	P		HR (95% CI)	P
Age					
≤72	Reference			Reference	
≥73	2.391 (1.780–3.211)	<0.001		2.228 (1.511–3.287)	<0.001
Race					
Black	Reference			--	
White	0.937 (0.635–1.383)	0.743		--	
Other	1.137 (0.605–2.138)	0.690		--	
Marital status					
Married	Reference			--	
Single	1.659 (1.116–2.466)	0.012		1.196 (0.790–1.811)	0.397
Other	1.705 (1.235–2.354)	0.001		0.859 (0.584–1.263)	0.440
Laterality					
Left	Reference			--	
Right	0.908 (0.680–1.214)	0.516		--	
Grade					
I	Reference			Reference	
II	1.867 (0.424–8.214)	0.409		1.031 (0.230–4.611)	0.968
III	3.065 (0.760–12.366)	0.116		1.244 (0.300–5.153)	0.763
IV	5.123 (1.122–23.395)	0.035		1.756 (0.369–8.360)	0.479
8th AJCC T stage					
T1	Reference			Reference	
T2	3.546 (1.875–6.707)	<0.001		2.738 (1.418–5.290)	0.003
T3	11.382 (5.991–21.624)	<0.001		5.222 (2.313–11.789)	<0.001
T4	16.052 (8.256–31.210)	<0.001		5.454 (2.353–12.644)	<0.001
8th AJCC N stage					
negative	Reference			Reference	
positive	2.860 (2.130–3.840)	<0.001		2.454 (1.730–3.482)	<0.001
8th AJCC M stage					
M0	Reference			Reference	
M1	7.832 (5.149–11.911)	<0.001		2.125 (1.270–3.556)	0.004
Tumor size					
≤4.2	Reference			Reference	
4.3–6.7	3.308 (2.319–4.720)	<0.001		1.536 (0.933–2.530)	0.092
≥6.8	7.714 (5.436–10.947)	<0.001		2.440 (1.325–4.492)	0.004
Subtype					
Luminal A	Reference			--	
Luminal B	1.141 (0.452–2.882)	0.780		--	
HER2 enriched	0.620 (0.246–1.566)	0.312		--	
Triple negative	1.117 (0.791–1.576)	0.531		--	
ER					
Positive	Reference			--	
Negative	1.195 (0.820–1.742)	0.354		--	
PR					
Positive	Reference			--	
Negative	1.066 (0.699–1.625)	0.766		--	
HER2					
Positive	Reference			--	
Negative	1.350 (0.714–2.554)	0.356		--	
Radiation					
No/unknown	Reference			Reference	
Yes	0.528 (0.391–0.713)	<0.001		0.518 (0.363–0.739)	<0.001
Chemotherapy					
No/unknown	Reference			Reference	
Yes	0.603 (0.450–0.809)	0.001		0.623 (0.430–0.903)	0.012
Surgery type					
Lumpectomy	Reference			Reference	
Mastectomy	3.129 (2.180–4.489)	<0.001		1.035 (0.683–1.567)	0.872

HR, hazard ratio; CI, confidence interval.

### To evaluate the prediction accuracy of TNM staging system and nomogram for overall survival

**FIGURE 3:**
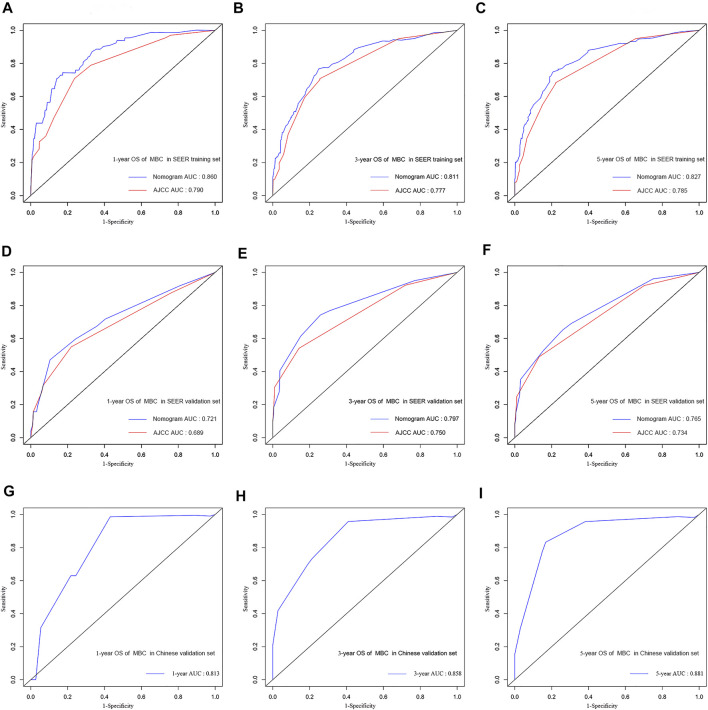
Comparison of the AUC of the nomograms and the eighth AJCC TNM staging system at 1, 3, and 5 years in the SEER training set **(A,B,C)** and in the SEER validation set **(D,E,F)**. ROC curves and AUC for nomograms at 1, 3, and 5 years in the multicenter validation set **(G,H,I)**.

**FIGURE 4:**
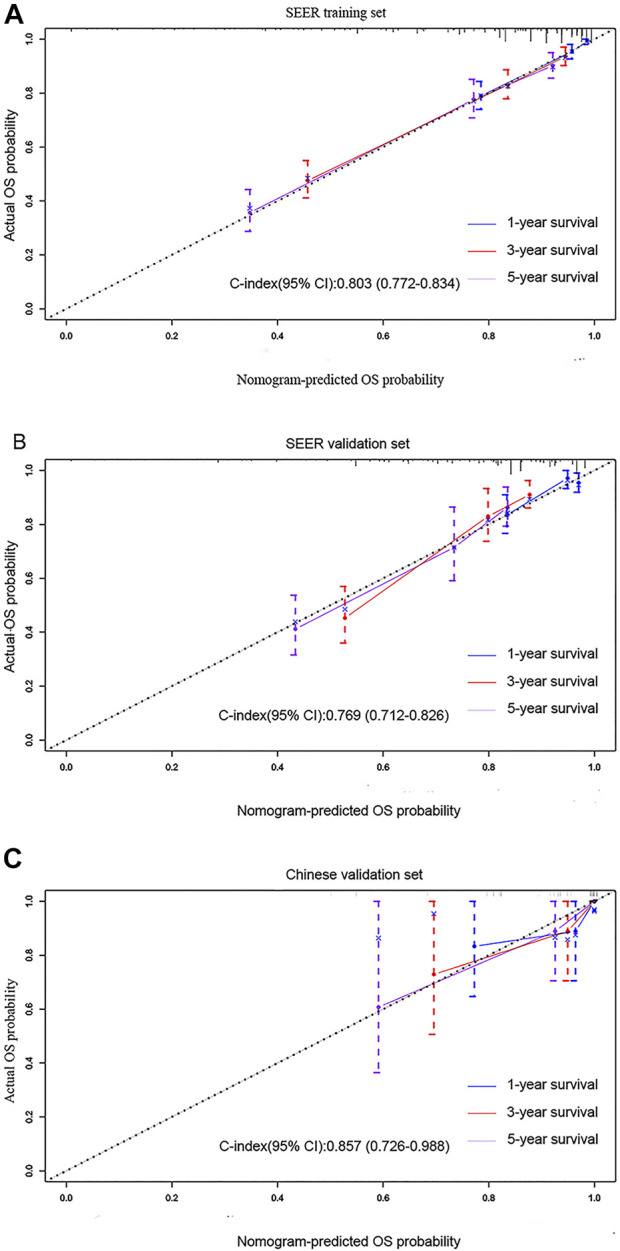
Calibration curves predicting the 1-, 3-, and 5-years OS of patients in the **(A)** training cohort, **(B)** SEER validation set, and **(C)** multicenter validation set. The *x*-axis plots the predicted survival probability, and the *y*-axis indicates the actual survival probability. The 45-degree inclined curve indicates that the predicted probability is in line with the actual probability.

In the training cohort and validation cohort of SEER, the C-index of the nomogram was 0.803, 0.769, which was higher than that of the AJCC staging system 0.752, 0.717. In addition, the AUCs confirmed the superiority of the predictive model for predicting the 1-year, 3-years, and 5-years survival in the training cohort (nomogram vs AJCC, 0.860 vs 0.790; 0.811 vs 0.777; 0.827 vs 0.785, respectively) (Figures 3A–C) and validation set (nomogram vs AJCC, 0.721 vs 0.689; 0.797 vs 0.750; 0.765 vs 0.734, respectively) (Figures 3D–F). The discrimination and survival prediction performance of the training set and internal verification set were improved compared with the eighth edition of the AJCC TNM. The Chinese validation set C-index was 0.857 (95% CI, 0.726–0.988). The AUC values for 1 year, 3 years, and 5 years were 0.813, 0.858 and 0.881, respectively (Figures 3G–I). The external verification set also proves that the nomogram has good distinguishing ability and predictive ability. In addition, the calibration curve test showed good agreement between the predicted values of the nomogram and the actual observed results (Figures 4A–C).

### Survival risk classification based on The Kaplan-Meier curve

The Kaplan-Meier curve showed that there were significant differences in OS among the different risk subgroups of the SEER training set (*p* < 0.001, Supplementary Figure S2A), which was confirmed in two validation data sets (SEER validation set, *p* < 0.001 Supplementary Figure S2B; multicenter validation set, *p* = 0.0158 Supplementary Figure S2C). Among all 1,044 patients, the nomogram showed great potential to differentiate between high- and low-risk groups (*p* < 0.001, Supplementary Figure S2D). In addition, in the training cohort, a subgroup analysis was performed to determine whether MBC was triple negative or not and the results showed that the differences were not significant (*p* = 0.338, Supplementary Figure S2E). Radiation therapy was found to provide a significant survival benefit in older women (*p* = 0.0052, Supplementary Figure S2F).

### Clinical application of the prognostic nomogram

The 1-, 3-, and 5-years DCAs in the nomogram showed greater net benefits than either “full treatment” or “no treatment” and performed better than the AJCC stage model, which demonstrates potential clinical applicability of the nomogram (Supplementary Figure S2G–I).

## Discussion

Conventional therapeutic approaches in MBC include surgery,chemotherapy, and radiation therapy. Surgery continues to be the standard therapy, in most case series. Due to the low incidence of MBC, it is difficult to collect clinical and pathological data; therefore, we constructed a nomogram for predicting postoperative survival of MBC patients based on data from a large-scale database in the United States, and verified its performance using data from multiple Chinese centers. The AJCC staging system takes into account only tumor size and location, local lymph node invasion, and distant metastases (Pezzi et al., 2007), and its ability to predict postoperative survival in MBC patients is poor. The nomogram combines factors including age, tumor size, and treatment information to evaluate the prognosis of patients. As far as we know, this is the first nomogram for predicting the postoperative survival of patients with MBC, and it can be used to provide patients with personalized services.

There are great differences between MBC and IDC in clinicopathological and biological aspects (Pezzi et al., 2007). Compared with IDC, the OS rate of MBC was lower (Paul Wright et al., 2014). In this study, seven factors were identified through univariate and multivariate analyses that were significantly related to the OS of patients with MBC, including age, T stage, N stage, M stage, tumor size, chemotherapy, and radiotherapy. T stage had the greatest impact on the survival of patients with MBC, and most patients had T2 stage disease. Many previous studies have also reported that the T stage of patients with MBC is higher than that of invasive ductal carcinoma. And compared with IDC, MBC is mostly blood metastasis. In addition, MBC is more likely to have lung and central nerve metastasis, while IDC has more bone metastasis (Song et al., 2013). The expression of HG and Ki-67 in MBC is higher than that in IDC (Jung et al., 2010). Therefore, it might not be appropriate to treat patients with MBC according to a management plan for IDC. This emphasizes the importance of the nomogram for informing personalized services for patients with MBC after surgery.

A retrospective analysis of 51 MBC patients found that triple-negative MBC is a favorable prognostic marker for patients with MBC. Their study also mentioned that TNMC is more prone to distant metastasis than NTNMC; however, after distant metastasis TNMC progresses more slowly, which may have caused the difference between the two subgroups (Lim et al., 2010). However, the differences between molecular typing of subtypes were not found to be statistically significant in our study. When subtypes were grouped into TNMC and NTNMC groups, there was no significant difference in survival between the two groups (*p* = 0.338). Previous studies have also shown this same result (Ong et al., 2018). Since their retrospective study had a small sample of individual institutions, the mechanism behind this result needs to be further explored.

Histology of MBC contains eight types after screening, including, metaplastic carcinoma, carcinosarcoma, squamous cell carcinoma, spindle cell carcinoma, sarcomatoid carcinoma, fibromatosis-like metaplastic carcinoma, low-grade adenosquamous carcinoma, and metaplastic carcinoma with chondroid differentiation or osseous differentiation (Hennessy et al., 2006; Zhang et al., 2021). When the data were grouped in a 7:3 ratio in this study, not all subtypes were included in the training set, and they were analyzed based on the whole population cohort. A univariate Cox regression analysis showed that histology was not a risk factor for OS after surgery in patients with MBC (*p* = 0.139). Previous studies also find no significant correlation between histology and prognosis (Leyrer et al., 2017; Corso et al., 2021).

Previous studies found that non-tumor related factors also has an impact on this disease (Ong et al., 2018). Univariate analysis showed that marital status is a risk factor for the prognosis of MBC in our study. Studys found that there was a striking relation to being overweight or obese, as well as the racial distribution of MBC occurrence. Additionally, results from their patient population also suggest that African American women with MBC tend to be overweight or obese, and may have worse OS compared with women of other ethnicities. These emphasizes that we might need to consider to the effect of non-biologic stressors on the disease in the future. However, additional multicenter studies are needed to further characterize these findings (Abada et al., 2022).

This study found that hormone receptor status (ER/PR) is not a risk factor that affects the survival of patients with MBC, which is consistent with the results of previous studies. Hormone therapy might not provide survival benefits to patients with MBC(Pezzi et al., 2007; Paul Wright et al., 2014). While chemotherapy was found to provide a survival benefit in patients with MBC after surgery (Lien et al., 2007; Reddy et al., 2020), it has a low impact. Studies showed that the poor prognosis and poor response to cytotoxic therapy of MBC suggested chemoresistance associated to epithelial-to-mesenchymal transition (EMT) and stem cell-like characteristics (Osako et al., 2009; Taube et al., 2010; Shah et al., 2012; Zhang et al., 2012). Several *in vitro* studies have demonstrated that resistance to anthracyclines and taxanes—the two types of drugs usually used in TNBC—is, at least in part, mediated by EMT (Gonzalez-Martinez et al., 2021). Zhang et al. provide evidence that epithelial to mesenchymal transition inducers and tumor initiating cells are present specifically in the non-glandular components of metaplastic carcinomas. Their study shows that metaplastic carcinomas of the breast have histological evidence and express biological markers of an epithelial to mesenchymal transition characterized by progressive overexpression of ZEB1 and downregulation of E-cadherin. This change in morphology and protein expression pattern is associated with an acquisitionof breast cancer stem cell marker proteins ALDH-1 and CD44^+^/CD24^−/low^, which may play distinct functional roles in metaplastic carcinomas (Zhang et al., 2012). Joneja et al. used the first-generation gene sequencing method to compare MBC, triple-negative breast cancer, and HER2-positive and hormone-positive breast cancer, and found that the expression rate of PD-L1 in MBC was higher than that of the other three cancer subtypes (46% vs 9, 6, 6%, respectively; *p* < 0.001) (Joneja et al., 2017). Although the expression mechanism is not yet clear, this provides a theoretical basis for the treatment of MBC using the immune system (Haque and Teh, 2018; Liu et al., 2022). Many mutated genes have been detected in MBC, and the most frequency mutated genes, TP53 and PIK3CA, suggest a direction for investigating targeted therapy (Tray et al., 2019; Gonzalez-Martinez et al., 2020).

In this study, the type of surgery had nothing to do with the prognosis of MBC patients, which is consistent with previously reported results (Corso et al., 2021). However, different studies suggest that surgical removal of the tumor has a great benefit if combined with radiation therapy, and an improvement of overall survival has been observed for both mastectomy and lumpectomy patients (Gadaleta-Caldarola et al., 2021). Li et al. showed that even elderly women (≥60 years old) can benefit from radiotherapy (Li et al., 2019). We found that even older female patients (≥73 years old), postoperative radiotherapy can also obtain significant survival benefits (*p* = 0.0052). At the same time, A recent study divided patients into low-risk (T1N0M0), intermediate-risk (T1-2N1M0 and T3N0M), and high-risk groups (T1-4N2-3M0 and T4N0-1M0), post-mastectomy radiotherapy (PMRT) could improve BCSS compared with that in non-PMRT patients in the high-risk groups; it also improved OS in both the intermediate- and high-risk groups (Hu et al., 2021). Indeed, because these studies had small sample sizes or were retrospective, caution is still needed when treating patients with reference to these studies. The development of standardized radiotherapy guidelines based on prospective studies with sufficient sample size would be of great clinical value.

Based on the results of the analysis, we constructed a nomogram to predict the postoperative survival of patients with MBC. The C-index and ROC curve of the nomogram performed better than the eighth edition of the AJCC TNM staging system. The calibration curve shows that the 1-year, 3-years, and 5-years survival rates predicted by the nomogram are similar to the actual survival rates. The nomogram also has good clinical applicability. However, due to the rarity of the disease and the small amount of data in the external validation set, the external data set did not validate that the nomogram performs better than the AJCC stage. Nonetheless, since the 40 cases of MBC from the large hospitals in China are representative of the real-world MBC situation, the nomogram is suggested to be applicable for practical use.

The study has some limitations. First, some patients were excluded due to incomplete information when the data was screened, and some selection bias may exist. Second, some important parameters and specific information related to prognosis, such as the family history of breast cancer, vascular invasion, chemotherapy regimens, and targeted therapies, are missing from the SEER database. Third, this is a retrospective study based on SEER and needs to be validated in a prospective clinical trial.

## Conclusion

In conclusion, the nomogram developed in this study is capable of predicting postoperative OS in patients with MBC. Validation of the internal dataset showed that the nomogram has good discrimination, predictive ability, and clinical practicability. The performance of the nomogram was further verified in the real world. These results suggest that the nomogram can help clinicians to predict postoperative OS in MBC patients and provide personalized services more accurately.

## Data Availability

The original contributions presented in the study are included in the article/Supplementary Material, further inquiries can be directed to the corresponding author.

## Abbreviations

metaplastic breast cancer (MBC); overall survival (OS); Surveillance, Epidemiology, and End Results Program (SEER); Harrells consistency index (C-index); the World Health Organization (WHO); estrogen receptor (ER); progesterone receptor (PR); human epidermal growth factor receptor 2 (HER2); invasive ductal cancer (IDC); the American Joint Committee on Cancer (AJCC); hazard ratio (HR); confidence interval (CI); receiver operating characteristic curve (ROC); area under the curve (AUC); triple negative metaplastic breast cancer (TNMC); non-triple negative metaplastic breast cancer (NTNMC); decision curve analysis (DCA); interquartile range (IQR).
