# Supplementary figures and images for: Establishment of prognostic model for postoperative patients with metaplastic breast cancer: Based on a retrospective large data analysis and Chinese multicenter study
Source: Front Genet. 2022 Aug 25;13:993116. doi: 10.3389/fgene.2022.993116 (PMC9454815; doi:10.3389/fgene.2022.993116)

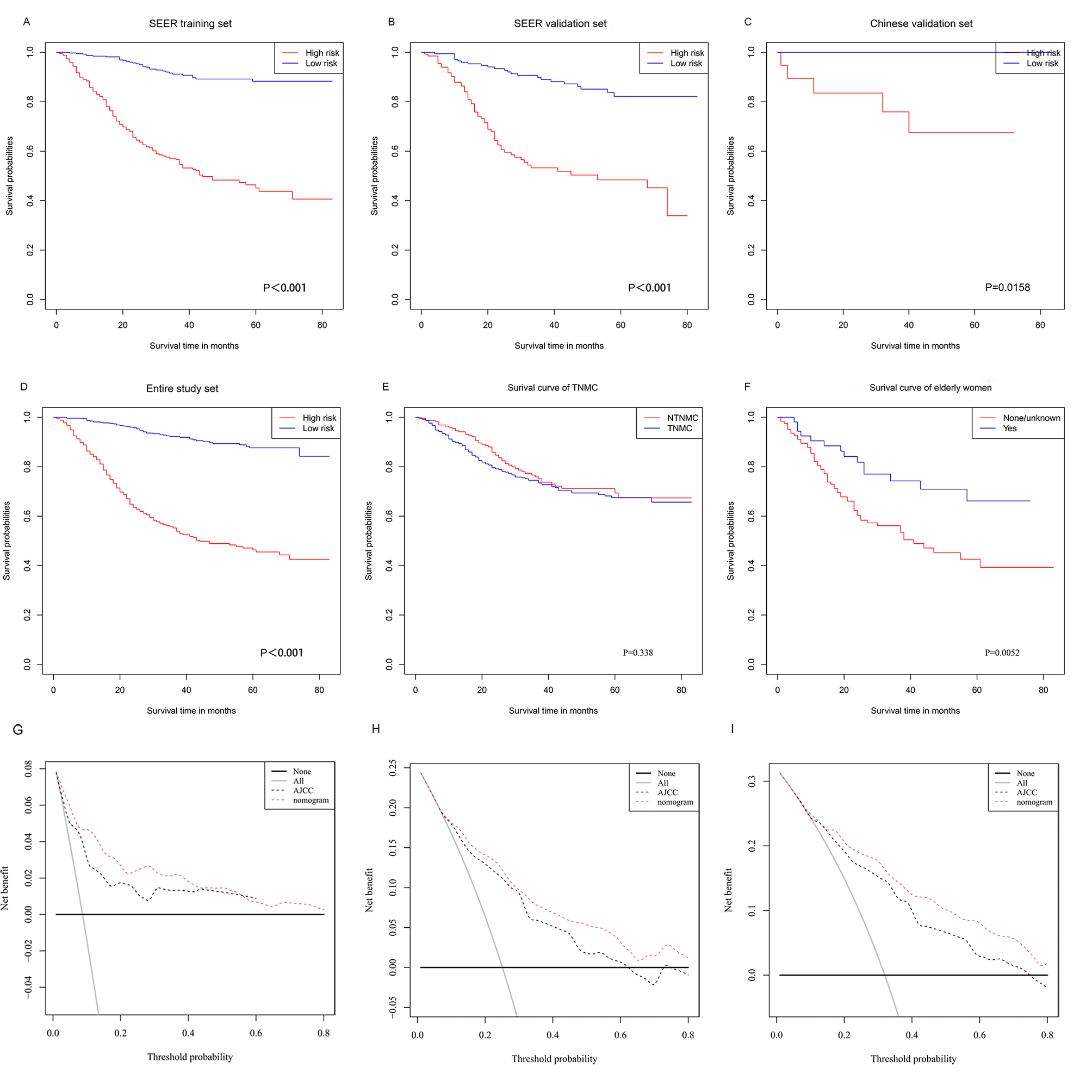

Supplement: Supplementary file 1 [file Image2.TIF]

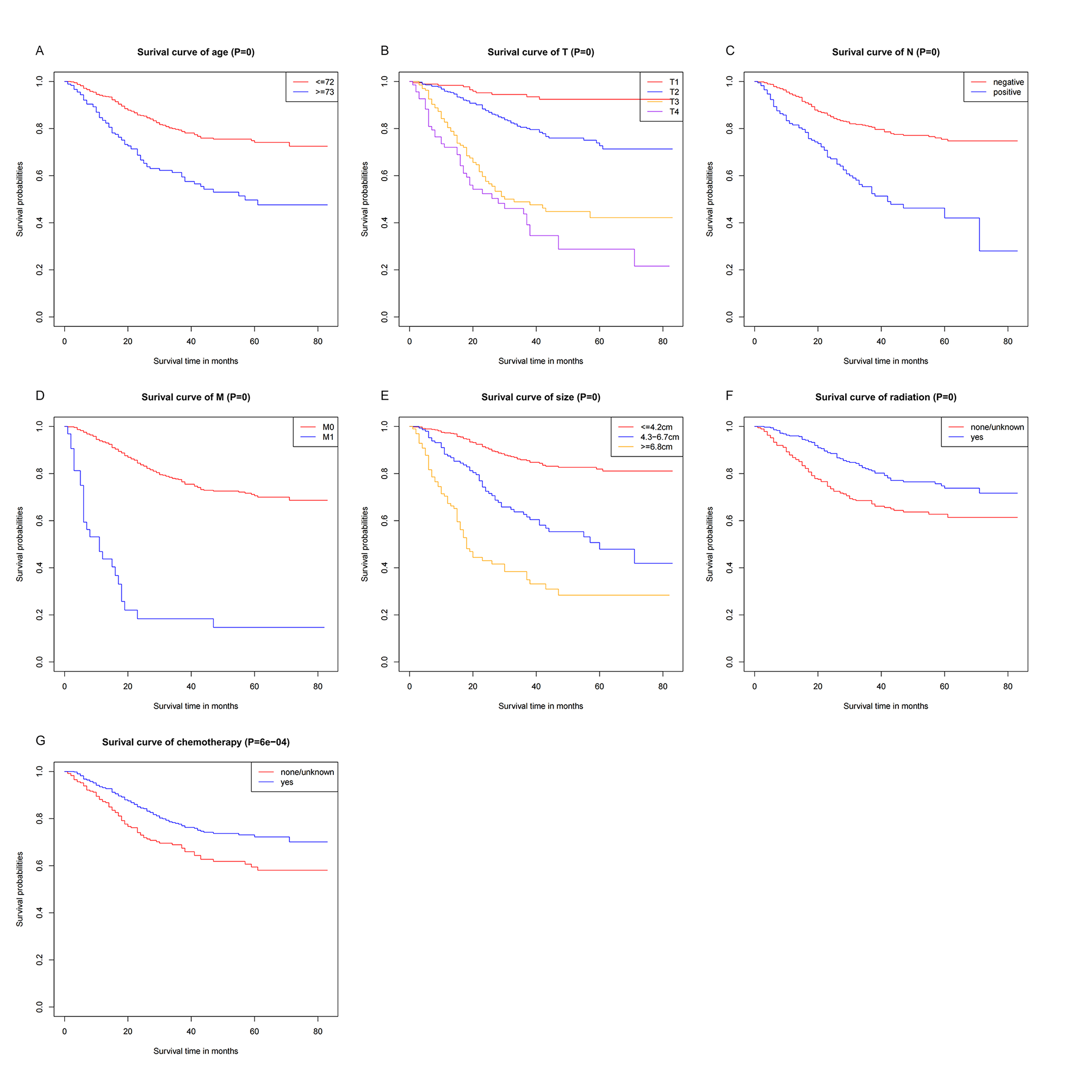

Supplement: Supplementary file 2 [file Image1.TIF]
